# Does Respiratory Rate Increase Before Respiratory Exacerbations in Pediatric Neuromuscular Patients Requiring Ventilation?

**DOI:** 10.1002/ppul.71592

**Published:** 2026-03-30

**Authors:** Sergio Ghirardo, Simona Basilicata, Barbara Madini, Alessandro Amaddeo

**Affiliations:** ^1^ Department of Medical, Surgical and Health Sciences University of Trieste Trieste Italy; ^2^ Institute for Maternal and Child Health IRCCS “Burlo Garofolo” Trieste Italy; ^3^ Department of Translational Medical Sciences, Paediatric Pulmonology Federico II University Naples Italy; ^4^ Pediatric Pneumonology Foundation IRCCS Cà Granda Ospedale Maggiore Policlinico Milan Italy

To the Editor,

In adult patients with chronic obstructive pulmonary disease (COPD) causing end‐stage chronic respiratory insufficiency, the use of long‐term noninvasive ventilation (NIV) can improve the outcomes of acute exacerbations [1]. In these patients, the analysis of the built‐in software of home ventilators shows an increase in respiratory rate (RR) starting from at least 2–3 days before a respiratory exacerbation. This variation can be helpful to predict a worsening of the respiratory conditions [2].

In this letter, we present our study, which evaluated RR as a predictor of respiratory exacerbation in a group of pediatric patients with neuromuscular disease (NMD) and chronic respiratory failure in long‐term NIV.

We retrospectively evaluated the built‐in software data from patients who experienced at least one exacerbation in the previous 12 months.

Exacerbations were defined by the presence of fever and/or an increase in upper airway secretions and/or SpO_2_ < 95% in room air on usual ventilatory settings, a prescription of an antibiotic for acute chest infection, or an unscheduled hospitalization for acute respiratory disease.

A total of 11 patients were included (six males and five females), with a median age of 5.3 years (Q1 3.5, Q3 16, range 2–19). Seven patients had spinal muscular atrophy type 1 (SMA1), four patients had spinal muscular atrophy type 2 (SMA2), four patients were affected by other myopathies, and one patient was affected by Steinert dystrophy. All the patients provided a written consent to retrospective data analysis of their clinical chart and to participate in retrospective studies such as this one.

Sixteen total exacerbations were recorded. Three patients had more than one exacerbation: one patient experienced two exacerbations, and two patients had three episodes. In three episodes (two in the same patient), the patients used the ventilator during the daytime, but only on the day of presentation; in the preceding days, all the patients had used it exclusively during the night, as usual. Of the 16 exacerbations, 14 were associated with increased upper airway secretions at presentation; 11 required hospitalization, 6 developed oxygen desaturation, 4 had fever, and 12 were started on antibiotics on the day of presentation.

For each patient, we extrapolated built‐in software data from their NIV devices, analyzing the type of ventilator used, mode of ventilation, type of interface, and ventilator settings.

Nine patients were using an Astral 150 (ResMed), one had a Trilogy 100 (Philips Respironics), and one was using a Trilogy EVO (Philips Respironics). Seven patients were ventilated using a spontaneous/timed (S/T) mode, four patients were ventilated with average volume‐assured pressure support (AVAPS), and one with average volume‐assured pressure support‐automated expiratory positive airway pressure (AVAPS‐AE). Ten patients were using a nasal mask and one an oronasal mask. Pressure was set with a median inspiratory positive airway pressure (IPAP) of 15.4 cmH_2_O (SD 1.16, Q1 15, Q3 16, range 14–18). Patients using an AVAPS mode had a median minimum IPAP of 13 (range 12–20) cmH_2_O and a median maximum IPAP of 17.5 (range 16–24) cmH_2_O, respectively. Median expiratory positive airway pressure (EPAP) was 5.5 cmH_2_O (SD 1.3, Q10, Q3, range 4–8 cmH_2_O). Patient ventilated with AVAPS‐AE mode had a minimum EPAP of 4 cmH_2_O, and a maximum EPAP of 12 cmH_2_O. Median set tidal volume was 105 milliliters (range 90–120) equal to median 9.0 mL/kg (7–10 mL/kg). Median backup rate was 18 breaths per minute (SD 4.0, Q1 15, Q3 20, range 14–28). All patients included in the study routinely use ventilation during nighttime, and this was the case on all days preceding the day of presentation. In three episodes (two in the same patient), patients also used the ventilator during daytime but exclusively on the day of presentation. Mean duration of hospitalization was 12 days, median 6 (SD 20, Q1 1, Q3 15, range 0–77). We defined the baseline as the data recorded by the device during the 30‐day period ending 6 days before the patient's clinical presentation. For the detailed data of the various parameter evolution during the 5 days prior to the day of the exacerbation, please refer to Table 1.

**Table 1:** Comparison of mean respiratory rate (RR) and range values over the 5 days prior to exacerbation.

	Basal data	5 days prior	4 days prior	3 days prior	2 days prior	1 day prior	Day of the exacerbation	*p* values ANOVA; basal versus day of exacerbation
RR (breaths/min)	19 (14–31)	19 (14–33)	20 (14–34)	21 (14–39)	21 (14–45)	22 (14–45)	22 (14–40)	0.74; 0.21
RR percentiles	21 (3–66)	22 (3–74)	26 (3–78)	31 (3–96)	31 (3–90)	38 (4–62)	39 (3–95)	0.39; 0.09
RR 95°centile (breaths/min)	27 (16–61)	27 (16–58)	28 (16–62)	29 (16–55)	29 (16–56)	31 (15–61)	32 (16–59)	0.88; 0.31
RR 95°centile percentiles	43 (9–99)	50 (26–78)	55 (10–99)	64 (10–99)	58 (10–99)	68 (5–99)	66 (10–99)	0.20; 0.049
Tidal volume (L)	255 (80–504)	259 (78–504)	245 (80–492)	249 (79–522)	237 (75–480)	242 (73–546)	231 (21–408)	0.99; 0.6
Median Ti (s)	1.00 (0.32–1.29)	1.00 (0.32–1.29)	0.99 (0.78–1.29)	1.00 (0.78–1.29)	0.98 (0.78–1.26)	0.92 (0.72–1.2)	0.96 (0.69–1.2)	0.39, 0.52
Mean leaks (L)	12 (0–35)	14 (0–35)	12 (0–36)	16 (0–37)	9 (0–37)	11 (0–35)	16 (0–38)	0.99; 0.67

*Note:* Basal data average of the 36 to 6 days before exacerbation; 5 days prior to the exacerbation, 4 days prior to the exacerbation, 3 days prior to the exacerbation, 2 days before the exacerbation, 1 day before the exacerbation.

Abbreviations: RR, respiratory rate; Ti, inspiratory time.

We used Bonafide et al. as a reference to calculate the RR percentile for each patient on every day of evaluation [3]. None of the parameters considered in the study changed statistically significantly comparing the days before presentation (–1 to −5) with the baseline. On the day of clinical presentation, the median RR percentile was the same as the baseline 95th percentile. The increase in the 95th percentile is statistically significant when comparing the baseline with the day of presentation, whereas the median RR percentile, although increasing over the days, does not show statistical significance.

Patients with a diagnosis of SMA1 presented a baseline median RR of 16 (Q1 14, Q3 19, range 14–28), with nearly no increase the day of the exacerbation: median increase 0 (Q1 0, Q3 1, range 0–3); *p* = 0.85; variance analysis *p* value through ANOVA test considering the baseline and all the days registered *p* = 0.99. On the other end, patients with dystrophies and myopathies presented a baseline median RR of 22 (Q1 18, Q3 27, range 18–31), with an increase the day of the exacerbation: median increase 7 (Q1 2.5, Q3 9.5, range 0–10); *p* = 0.19; variance analysis *p* value through ANOVA test considering the baseline and all the days registered *p* = 0.73. This increase was not observed in patients with SMA1, even though trigger settings were sensitive or very sensitive in most cases. These settings did not differ from those applied in patients with myopathies. To appreciate the difference in RR between SMA and myopathies, please refer to Figure 1 reporting in white patients with SMA and in blue patients with myopathies.

**Figure 1:**
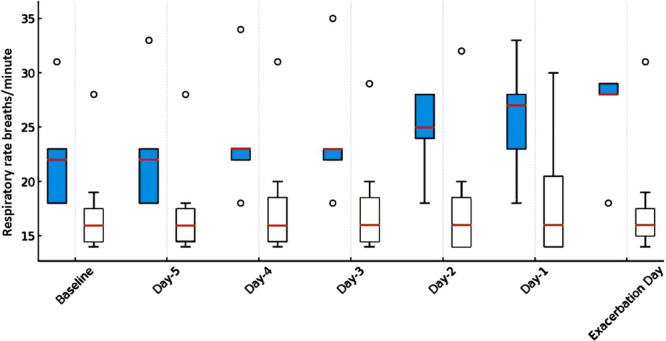
Respiratory rate variation at baseline, in the 5 days before exacerbation, and on the day of exacerbation in patients with SMA (white boxes) and patients with other neuromuscular conditions (blue boxes).

In conclusion, our pilot study conducted on a limited population of pediatric patients with NMD analyzed the RR as a potential predictive factor for respiratory exacerbation. Contrary to what is observed in adult COPD patients, RR does not appear to be a reliable predictor of respiratory exacerbation in this population. This difference may be due to the reduced muscular strength typical of NMD patients, but also due to the small sample size as the percentile of the 95°centile RR showed a slightly significant increase.

However, an increase in RR was observed on the very day of clinical presentation in patients with myopathies and Steinert's disease, who may have relatively better‐preserved muscular strength compared to other NMD subtypes, with the ability to increase RR as a compensation during exacerbation. A similar, though not statistically significant, trend in RR was also observed in this group during the 2 days preceding clinical presentation.

Further studies on a larger cohort, with more detailed assessments of respiratory muscular strength, are necessary to confirm our results.

## Author Contributions

**Sergio Ghirardo:** conceptualization, data collection, data analysis and draft writing. **Simona Basilicata:** data collection and draft writing. **Barbara Madini:** data collection, proof evaluation. **Alessandro Amaddeo:** conceptualization and proof evaluation.

## Funding

The authors have nothing to report.

## Ethics Statement

Ethical Committee approval was not requested according to the Italian Law since General Authorization to Process Personal Data for Scientific Research Purposes (Authorization no. 9/2014) declared that retrospective archive studies that use ID codes, preventing the data from being traced back directly to the data subject, do not need ethics approval: The Italian Data Protection Authority. Available at: https://www.garanteprivacy.it/web/guest/home/docweb/-/docweb-display/docweb/3786078 (Accessed March 2025). Authorization no. 9/2014‐General Authorization to Process Personal Data for Scientific Research Purposes.

## Conflicts of Interest

The authors declare no conflicts of interest.

## Data Availability

The data that support the findings of this study are available on request from the corresponding author. The data are not publicly available due to privacy or ethical restrictions.
